# Environmental Stressors, Anemia, and Depressive Symptoms in Pregnancy: Unpacking the Combined Risks

**DOI:** 10.3390/ijerph22111727

**Published:** 2025-11-15

**Authors:** Ruth A. Pobee, Rebecca K. Campbell, Prathiba Balakumar, Yongchao Huang, Beatriz Peñalver Bernabé, Mary Dawn Koenig

**Affiliations:** 1 Department of Emergency Medicine, College of Medicine, University of Illinois Chicago, 808 S Wood St., Chicago, IL 60612, USA; 2 Division of Epidemiology and Biostatistics, University of Illinois Chicago, 1603 W Taylor St., Chicago, IL 60612, USA; rcampb@uic.edu (R.K.C.); pbalak3@uic.edu (P.B.); 3 Department of Biomedical Engineering, Colleges of Engineering and Medicine; University of Illinois Chicago, 851 S Morgan St., Chicago, IL 60607, USA; yhuan66@uic.edu (Y.H.); penalver@uic.edu (B.P.B.); 4 Center for Bioinformatics and Quantitative Biology, University of Illinois Chicago, 820 S Wolcott St., Chicago, IL 60612, USA; 5 Department Human Development Nursing Science, College of Nursing, University of Illinois Chicago, 845 South Damen Avenue, Chicago, IL 60612, USA; marydh@uic.edu

**Keywords:** maternal depressive symptoms, anemia, environmental stressors, health disparities

## Abstract

Chronic exposure to structural violence and environmental hazards may disrupt stress regulation, trigger inflammation, and impair iron metabolism in women. Iron deficiency has been associated with depression, but the combined impact of environmental stressors and anemia on maternal mental health remains understudied. We analyzed associations between 28 neighborhood-level environmental stressors, hemoglobin levels, and depressive symptoms (measured by the Patient Health Questionnaire-9) during early pregnancy, using retrospective data from 1964 pregnant patients (2015–2019) at an urban health center in Chicago. Demographic and residential data were linked to environmental indicators from the Chicago Health Atlas. Factor analysis reduced the environmental variables, and multivariable regression models examined associations with PHQ-9 scores at first pregnancy encounter. Participants were predominantly non-Hispanic Black (56%) and Hispanic (27%), with 13% anemic and 16% screening positive for depressive symptoms. Poverty, non-Hispanic Black race, single status, public or no insurance, and unemployment were associated with higher depressive symptoms. Among anemic individuals, neighborhood crime was significantly associated with depressive symptoms, while hemoglobin levels and gestational age were not. These findings highlight how environmental and social inequities contribute to maternal mental health disparities and support the need for integrated, equity-focused prenatal care interventions.

## 1. Introduction

Antenatal depression is a significant public health concern that affects up to 25% of pregnant individuals in high-income countries, including the United States [1,2]. It is often underdiagnosed because its symptoms, such as fatigue, appetite changes, and sleep disturbances, overlap with common pregnancy experiences [3]. Left untreated, antenatal depression can result in serious complications, including preterm birth, low birth weight, hypertensive disorders, and poor maternal self-care [4,5,6]. Studies have also shown associations between antenatal depression and unhealthy coping behaviors like smoking, consuming alcohol, or drug use, which further increase risks for both the mother and fetus [7]. Importantly, antenatal depression increases the likelihood of postpartum depression, potentially impairing maternal–infant bonding and caregiving [8]. Children born to mothers with antenatal depression face higher risks of behavioral problems, developmental delays, and emotional challenges, underscoring the need for early identification and intervention [9].

Iron deficiency anemia, a common pregnancy complication, has been increasingly linked to depressive symptoms. Iron plays a crucial role in neurotransmitter synthesis, including dopamine and norepinephrine, which regulate mood and cognitive function [10,11,12]. Iron deficiency may contribute to depression through fatigue, low energy, and neurochemical dysregulation [13,14]. Among pregnant individuals, anemia has been associated with elevated risks for both antenatal and postpartum depression [15].

Both anemia and depression are shaped by environmental factors and social determinants of health. Low socioeconomic status, food insecurity, and limited healthcare access heighten the risk of iron deficiency anemia, particularly among racial and ethnic minority groups facing systemic inequities [16]. Chronic stress, stemming from structural violence such as discrimination and neighborhood disadvantage, further disrupts iron metabolism through stress-induced inflammation and elevated hepcidin levels, which impair iron absorption and storage [17,18]. Environmental factors, including poverty, food deserts, and exposure to pollutants, can also compromise nutritional status and hemoglobin production, exacerbating anemia risk [16,19].

While both anemia [15] and environmental stressors [20] have been individually associated with depression, little is known about their combined impact on maternal mental health during pregnancy. In particular, the role of environmental exposures in shaping depression risk among pregnant individuals with anemia remains underexplored. This study aims to investigate the interplay between environmental stressors, maternal iron status, and depressive symptoms during first pregnancy encounter, in an urban setting characterized by high-risk for adverse maternal health outcomes.

## 2. Methods

### 2.1. Study Design and Data Sources

This retrospective cohort study used electronic health records from pregnant women aged 18–55 who received prenatal care between 2015 and 2019 at the University of Illinois, Chicago (UIC). Demographic data (age, race, ethnicity, marital status, employment status, insurance status, and residential address) were extracted. Clinical data included hemoglobin levels, body mass index (BMI), gestational age at the first prenatal visit, and depressive symptom scores. For patients with multiple pregnancies during the study period, only the first pregnancy record was included. Participants were excluded from the analyses if they lacked both PHQ-9 and hemoglobin data, had missing covariates, or lived outside the Chicago boundaries. All data were obtained with the support of the Center for Clinical and Translational Science (CCTS) at UIC.

### 2.2. Measures

Depressive symptoms were assessed using the Patient Health Questionnaire-9 (PHQ-9), categorized as low (<10) or high (≥10) based on standard cut-offs [21]. Anemia was defined as hemoglobin < 11 g/dL in the first trimester and <10.5 g/dL in the second trimester [22].

### 2.3. Environmental Exposure Data

Neighborhood level environmental variables were sourced from the Chicago Health Atlas [23], aligned with the 2016–2019 timeframe of patient records. A total of 28 variables were extracted, representing neighborhood-level exposures related to crime, socioeconomic status, food access, pollution, and housing. These included rates of violent crime, unemployment, food insecurity, eviction, lead exposure, and neighborhood safety indicators. Full variable descriptions and years are provided in Supplemental Table S1.

### 2.4. Covariates

Covariates included race, ethnicity, marital status, employment status, insurance status, gestational age (approximated based on the last menstrual period), BMI, and hemoglobin level at first pregnancy encounter.

### 2.5. Statistical Analyses

Analyses were conducted using SAS version 9.4 (SAS Institute Inc., Cary, NC, USA). Descriptive statistics summarized participant characteristics. Group differences were assessed using the chi-square test for categorical variables and a *t*-test or a Wilcoxon rank-sum test for continuous variables, depending on distribution. The primary outcome variable was high depressive symptoms (PHQ-9 ≥ 10) at the first pregnancy encounter.

### 2.6. Exploratory Factor Analysis (EFA) of Environmental Variables

EFA was performed to reduce 28 correlated neighborhood-level variables into latent constructs [24]. Suitability for factor analysis was confirmed using the Kaiser–Meyer–Olkin (KMO) measure and Bartlett’s test of sphericity [25]. Principal axis factoring with promax rotation was applied. The number of factors retained was determined using a scree plot, parallel analysis, and minimum average partials (MAP) [26,27,28]. Variables with pattern loadings ≥ 0.30 were considered salient, with at least three salient items per factor required [29]. Factor internal consistency was evaluated using Cronbach’s alpha (≥0.70) [30]. Retained factors were interpreted based on theoretical coherence and percent variance explained.

### 2.7. Regression Analyses

Multivariable logistic regression models examined associations between environmental factors (factor scores) and depressive symptoms, adjusting for individual-level covariates. Models were constructed for the overall sample and stratified by anemia status (anemic vs. non-anemic). We ran both unadjusted and adjusted models for the full sample and separately for women with and without anemia. In the unadjusted models, each demographic and environmental factor was examined independently, while the adjusted models included all demographic covariates together with the retained environmental factors (crime, poverty, and pollution). Statistical significance was set at *p* < 0.05.

## 3. Results

Among the 1964 pregnant women included in the study, 70% were in their first trimester and 30% in their second trimester at the first recorded antenatal care visit. The mean age was 31.1 years (SD ± 6.2), with a median gestational age of 73 (IQR: 57–98). Over half of the women were non-Hispanic Black (56%), followed by Hispanic (28%), non-Hispanic White (9%), and non-Hispanic Asian/Other (4%). Most women (78%) were single, in a non-committed relationship, or had no marital status recorded, and 68% were unemployed, while 22% were employed, and 5% reported part-time employment or student status. Regarding insurance, 72% were not covered by private plans, while 28% had private insurance.

Obesity prevalence was 43%, and the mean hemoglobin levels were 12.2 g/dL (SD ± 1.2) overall, 12.4 g/dL (SD ± 1.2) from patients that had their first OB visit within the first trimester and 11.7 g/dL (SD ± 1.1) from those within the second trimester. Full sample characteristics by depressive symptom categories are presented in Table 1.

### 3.1. Environmental Factor Analysis

Exploratory factor analysis of the 28 community-level environmental variables yielded a three-factor solution comprising 23 items. Five variables—food insecurity, eviction rate, low food access, lead poisoning, and larceny—were excluded due to cross-loading (difference < 0.2). Together, the three factors explained 98.9% of the variance and demonstrated good reliability (Cronbach’s alpha, *α* = 0.84).

- Factor 1 (Crime Factor): Included indicators such as robbery, violent crime, assault, and drug abuse (*α* = 0.98).
- Factor 2 (Poverty Factor): Captured economic hardship indicators like unemployment, rent burden, and food stamps usage (*α* = 0.77).
- Factor 3 (Pollution Factor): Comprised crowded housing, air pollution, and limited green space (*α* = 0.95).

Details of factor loadings are presented in Table 2.

### 3.2. Predictors of Depressive Symptoms

In the total sample, higher poverty factor scores were associated with greater depressive symptoms at the first pregnancy visit (*β* = 0.24, *p* = 0.01); however, this association was no longer significant after adjusting for demographic and clinical covariates (*p* = 0.72). Among demographic factors, non-Hispanic Black women were more likely to report significantly higher depressive symptoms compared to non-Hispanic White women (*β* = 1.17, *p* = 0.01). Single women also exhibited higher depressive symptoms than their non-single counterparts, both before adjustment (*β* = 1.28, *p* = 0.01) and after adjusting for other demographic and environmental factors (*β* = 0.82, *p* = 0.01). Having private insurance was associated with lower depressive symptoms, with both unadjusted (*β* = −0.97, *p* = 0.01) and adjusted (*β* = −0.51, *p* = 0.04) models showing a statistically significant association. Unemployment was similarly linked to higher depressive symptoms, both independently (*β* = 0.80, *p* = 0.01) and in the multivariate model (*β* = 0.52, *p* = 0.04). No significant difference in depressive symptoms was observed by Hispanic ethnicity, Asian/Other race, hemoglobin levels, or gestational age, and there was no interaction between hemoglobin levels and demographic or neighborhood factors.

### 3.3. Subgroup Analyses

Among pregnant women with anemia (*n* = 258), higher crime factor scores were independently associated with greater depressive symptoms (*β* = 0.58, *p* = 0.02), and this association remained significant after adjusting for covariates (*β* = 0.65, *p* = 0.03). No other environmental factor, demographic characteristic, or hemoglobin level was significantly associated with depressive symptoms in this subgroup. In contrast, findings in the non-anemic sample (*n* = 1705) mirrored those of the total sample (*n* = 1963). Higher poverty factor scores were independently associated with increased depressive symptoms (*β* = 0.28, *p* = 0.01). Non-Hispanic Black women were more likely to report depressive symptoms compared to non-Hispanic White women (*β* = 1.22, *p* = 0.01). Single women also showed higher depressive symptoms, both before (*β* = 1.30, *p* = 0.01) and after adjusting for demographic and environmental factors (*β* = 0.28, *p* = 0.01). Private insurance was associated with lower depressive symptoms in both unadjusted (*β* = −0.03, *p* = 0.01) and adjusted models (*β* = −0.58, *p* = 0.03). Additionally, unemployment was linked to higher depressive symptoms (*β* = 0.73, *p* = 0.01). Results are in Table 3.

## 4. Discussion

This study examined the associations between neighborhood environmental stressors (crime, poverty, and pollution) and individual-level factors (demographic characteristics and hemoglobin levels) on maternal depressive symptoms during first pregnancy encounter. Using factor analysis, we distilled complex, interrelated neighborhood characteristics into three distinct dimensions: crime, poverty, and pollution, and incorporated these into multivariable models to explore contextual influences on maternal mental health. Our findings contribute to a growing body of literature that highlights the importance of environmental and social determinants in maternal mental health, while also shedding light on new relationships between neighborhood stressors and depressive symptoms in pregnant women, especially those with anemia.

In the overall sample and among non-anemic women, poverty emerged as the primary environmental stressor associated with elevated depressive symptoms. This finding is consistent with numerous studies linking economic insecurity to higher maternal depression [31,32,33]. Economic hardship, which often includes limited access to nutritious food, stable housing, quality prenatal care, and mental health services, compounds the stress experienced by pregnant women, thereby increasing their vulnerability to depressive symptoms. In a study by Katz et al., it was found that economic instability, including financial strain and lack of access to resources, was significantly correlated with higher rates of depression among pregnant women [34]. This supports our own findings, where community level poverty remained an independent predictor of depressive symptoms among non-anemic and overall sample.

Furthermore, our study underscores the role of demographic factors such as single status, unemployment, and lack of private insurance in contributing to depressive symptoms. These findings are consistent with previous literature that has highlighted the protective role of stable relationships [35,36,37,38], employment [39], and private insurance [40,41] in reducing maternal depression. For instance, a study by Robles et al. found that single mothers were more likely to experience depressive symptoms due to the compounded stress of caregiving responsibilities and economic insecurity [37]. Similarly, uninsured pregnant women are more likely to experience untreated mental health issues, which may exacerbate depressive symptoms [41]. Our study’s results, which show that single status, unemployment, and lack of private insurance were strongly associated with depressive symptoms, echo these findings and suggest that socioeconomic conditions may more directly impact maternal mental health than race alone, though structural inequities remain relevant.

Interestingly, our study found that non-Hispanic Black women were more likely to report significantly higher depressive symptoms compared to non-Hispanic White women. However, after adjusting for demographic and environmental factors, this association was no longer significant, while factors such as single marital status, lack of insurance, and unemployment remained significant predictors. This suggests that socioeconomic factors may have a stronger influence on maternal mental health than race itself, although it does not negate the fact that structural inequities and systemic racism continue to shape the lived experiences of Black women in the U.S. As demonstrated by Williams and Mohammed, racial disparities in health outcomes are influenced by historical and ongoing structural racism, which manifests in economic, social, and healthcare disparities [42]. Our study’s attenuation of the racial disparity in depressive symptoms may indicate that, while race plays a role in maternal mental health, it is intertwined with broader socioeconomic stressors that need to be addressed to improve outcomes for marginalized populations.

One of the more novel findings of our study was the distinct pattern observed among pregnant women with anemia. Specifically, crime-related neighborhood stressors emerged as the only significant environmental predictor of depressive symptoms in this subgroup. While no similar associations were observed in the overall or non-anemic samples, our results are in line with existing research on the link between neighborhood crime and maternal mental health. A study by Shannon et al. found that women living in high-crime neighborhoods had increased odds of experiencing depression during pregnancy, suggesting that crime-related stress can contribute to heightened emotional distress [43]. Our study adds to this literature by identifying a specific vulnerability among anemic pregnant women. This is noteworthy because iron deficiency anemia has been linked to impaired mood regulation and neurotransmitter metabolism [12], potentially exacerbating the psychological burden of living in high-crime areas [44,45]. These findings suggest that the combined impact of environmental stress and physiological conditions such as anemia may create a synergistic vulnerability to depression, warranting further investigation at multiple time points to confirm these findings.

Interestingly, hemoglobin levels themselves were not directly associated with depressive symptoms in any of our groups. This finding contrasts with studies that have suggested a direct link between iron deficiency anemia and depression [46,47]. For example, a study by Corwin et al. found that pregnant women with lower hemoglobin levels were more likely to experience depressive symptoms, while a review and meta-analysis by Azami et al. highlighted that anemia during pregnancy and after pregnancy significantly increased the risk of postpartum depression [48]. However, our results are consistent with other studies that have failed to find a direct association between hemoglobin levels and depressive symptoms [44]. This discrepancy may be due to a variety of factors, including differences in study populations, the timing of hemoglobin and/or depressive symptom assessment, and the role of other confounding variables, such as nutrition and psychosocial stress, which could influence both anemia and mental health outcomes. The lack of a direct relationship between hemoglobin and depressive symptoms in our study highlights the need for a more nuanced understanding of the factors that contribute to maternal depression, emphasizing the importance of considering broader contextual influences alongside physiological markers.

### Strengths and Limitations

A major strength of this study lies in its novel application of factor analysis to reduce the complexity of neighborhood-level environmental data. By condensing multiple interrelated environmental variables into three primary factors—crime, poverty, and pollution—we were able to examine the unique contributions of these stressors to maternal mental health. This methodological approach improves upon previous studies that may have considered individual environmental factors in isolation, offering a more holistic view of the cumulative impact of multiple related stressors and the interplay between stressor domains. Additionally, by examining both anemic and non-anemic subgroups, we were able to identify how environmental stressors differentially affect women with varying health profiles, thereby providing a more comprehensive understanding of maternal mental health risks.

However, there are several limitations to our study. First, the cross-sectional design precludes the establishment of causal relationships, and depressive symptoms may change over the course of pregnancy [49,50]. Longitudinal studies are needed to explore how environmental stressors and individual factors interact over time to affect maternal mental health. Second, the cause of anemia could not be determined due to limited iron and other hematological biomarker data. The lack of information on supplementation practices and comorbid medical conditions may also influence the observed results. Also, our reliance on data from a single health center serving primarily non-Hispanic Black patients, may limit the generalizability of our findings to broader populations. Further research in diverse settings is necessary to validate these results. Additionally, the use of self-reported depressive symptoms (PHQ-9) may introduce reporting bias, as individuals may underreport or overreport their symptoms based on social desirability or other factors. Finally, while we included several environmental and demographic factors in our analysis, there may be unmeasured confounders, such as nutritional status, trauma history, and chronic stress, that could influence depressive symptoms. In addition, not all environmental-level variables aligned precisely with the time frame of our dataset. This discrepancy is largely due to differences in data availability and reporting cycles, as many environmental indicators are released annually, aggregated across calendar years, or reported with a lag. As such, the measures used represent the best available approximations of environmental exposures during the study timeframe.

## 5. Conclusions

Our study underscores the critical role of poverty as a driver of depressive symptoms during pregnancy and highlights the unique impact of crime-related stress on anemic pregnant women. The findings also reinforce the importance of demographic factors such as single status, unemployment, and lack of private insurance in exacerbating maternal mental health risks. These results call for more research into the combined effects of physiological and environmental stressors during pregnancy, especially among marginalized populations exposed to structural violence. Clinically, our findings support a holistic approach to prenatal care that addresses both medical and social risk factors. Interventions targeting poverty reduction, improving neighborhood safety, and enhancing access to mental health care could significantly improve maternal well-being and pregnancy outcomes.

## Figures and Tables

**Table 1:** Distribution of patient demographics characteristics stratified by depressive symptoms, among UI Health pregnant patients, 2015–2019.

Demographic Variables	Depressive Symptoms *n* (%)			
	Total Sample	Low Depressive Symptoms, <10	Depressive Symptoms, ≥10	*p*-Value
	1964 (100%)	1656 (84%)	308 (16%)	
Trimester 1st 2nd	1365 (69.5) 599 (30.5)	1147 (69.3) 509 (30.7)	218 (70.8) 90 (29.2)	0.60
Age, years- mean (SD)	31.1 (6.2)	31.2 (6.1)	30.4 (6.5)	**0.03**
Gestational age, days-median (IQR)	73 (57–98)	73.0 (57–99)	72.5 (57–97)	0.89
Race and Ethnicity Hispanic Non-Hispanic White Non-Hispanic Black Non-Hispanic Asian Other race and ethnicity	537 (27.5) 172 (8.8) 1089 (55.8) 69 (3.5) 85 (4.4)	467 (28.4) 155 (9.4) 889 (54.1) 60 (3.7) 73 (4.4)	70 (22.8) 17 (5.5) 200 (64.9) 9 (2.9) 12 (3.9)	**0.01**
Marital Status Single * Not Single	1536 (78.2) 428(21.8)	1264 (76.3) 392 (23.7)	272 (88.3) 36 (11.7)	**<0.001**
Employment Employed Part-time Student Not Employed	422 (21.5) 101 (5.1) 113 (5.8) 1328 (67.6)	374 (22.6) 84 (5.1) 96 (5.8) 1102 (66.6)	48 (15.6) 17 (5.5) 17 (5.5) 226 (73.4)	0.05
Insurance Private ** Non-private	550 (27.9) 1425 (72.2)	485 (29.3) 1171 (70.7)	63 (20.4) 245 (79.6)	**0.002**
BMI, kg/m^2^ Obese ≥ 30 Non-Obese, <30	822 (42.6) 1109 (57.4)	700 (43.0) 927 (57.0)	122 (40.1) 182 (59.7)	0.35
Hemoglobin, g/dL median (IQR)	12.3 (11.5–13.0)	12.3 (11.3, 13.0)	12.2 (11.6, 13.0)	0.48

* Single: includes unmarried, separated, or divorced; Married: includes married or in a committed relationship. ** Private: includes private or commercial insurance; Non-private: includes non-commercial, government and un-insurance. *p*-value < 0.05 are in bold font.

**Table 2:** Pattern Coefficients and fit indices for the three-factor solutions from the 28 environmental level characteristics.

Environmental Characteristics	Crime	Poverty	Pollution	Communality (h^2^)
Robbery	**1.00652**	−0.08319	−0.00306	0.98951
Criminal sexual assault	**0.99725**	−0.12764	−0.03311	0.97017
Traffic crashes	**0.96975**	−0.34025	0.17484	0.93450
Violent crime	**0.95631**	0.12783	−0.07376	0.99178
Smoking during pregnancy	**0.93628**	0.14549	−0.01250	0.95003
Arson	**0.90107**	0.21589	0.10202	0.92823
Aggravated assault/battery	**0.89513**	0.25061	−0.11367	0.97378
Homicide	**0.87273**	0.31101	−0.05482	0.96807
Property crime	**0.84843**	−0.55520	−0.10114	0.88100
Drug abuse	**0.64144**	0.30198	0.11565	0.57860
Hardship index	−0.02238	**0.92950**	0.21575	0.91755
Social vulnerability index	0.11771	**0.89966**	0.18543	0.90467
Unemployment rate	0.02562	**0.84990**	−0.35071	0.83538
Food stamps	0.18494	**0.83981**	−0.29928	0.87815
Rent burdened	0.25119	**0.76969**	−0.00766	0.72702
Traffic intensity	0.09761	**−0.55192**	−0.10328	0.31027
Easy access to fruits and vegetables	−0.15134	**−0.67762**	−0.00843	0.52028
Neighborhood safety	0.56903	**−0.80165**	0.10729	0.79394
College graduation rate	0.02062	**−0.90707**	−0.27418	0.90906
Per capita income	−0.00222	**−0.95948**	0.01737	0.92059
Crowded housing	−0.00682	0.12751	**0.74377**	0.57634
Particulate matter (PM 2.5) concentration	0.17625	0.33825	**0.67022**	0.61377
Tree canopy	0.09822	0.18021	**−0.78366**	0.66517
**Fit Indices**				
Eigenvalue % Variance Cronbach’s Alpha	10.14 8.39 0.98	6.54 7.50 −0.77	2.06 2.08 −0.95	

The bolded pattern coefficients show the primary factor for each variable. Detailed description of environmental characteristics is described in Supplemental Table S1.

**Table 3:** Environmental, Health, and Demographic Predictors of Depressive Symptoms at First Pregnancy Encounter among UI Health pregnant patients, 2015–2019.

Environmental Factors and Patient Demographic Characteristics	Total Sample, n = 1963				Anemic Individuals, n = 258				Non-Anemic n = 1705			
	Unadjusted		Adjusted		Unadjusted		Adjusted		Unadjusted		Adjusted	
	Estimate (SE)	*p*-Value	Estimate (SE)	*p*-Value	Estimate (SE)	*p*-Value	Estimate (SE)	*p*-Value	Estimate (SE)	*p*-Value	Estimate (SE)	*p*-Value
Crime factor	0.17 (0.09)	0.07	0.01 (0.11)	0.97	0.58 (0.26)	**0.02**	0.65 (0.30)	**0.03**	0.10 (0.10)	0.31	−0.09 (0.11)	0.46
Poverty factor	0.24 (0.09)	**0.01**	0.04 (0.11)	0.72	−0.07 (0.28)	0.82	−0.14 (0.31)	0.64	0.28 (0.10)	**0.01**	0.08 (0.12)	0.50
Pollution factor	−0.17 (0.11)	0.08	−0.03 (0.11)	0.78	−0.07 (0.29)	0.82	0.05 (0.36)	0.89	−0.18 (0.10)	0.08	−0.06 (0.12)	0.64
Hemoglobin, g/dL	−0.04 (0.08)	0.61	0.12 (0.09)	0.19	0.17 (0.29)	0.55	0.21 (0.31)	0.51	−0.08 (0.12)	0.50	0.14 (0.13)	0.27
Gestational age days	0.01 (0.01)	0.80	−0.01 (0.01)	0.72	0.01 (0.01)	0.98	0.01 (0.01)	0.93	0.01 (0.01)	0.85	−0.01 (0.01)	0.81
BMI	−0.01 (0.01)	0.94	−0.01 (0.01)	0.41	−0.01 (0.01)	0.66	−0.02 (0.03)	0.51	0.01 (0.01)	0.93	−0.01 (0.01)	0.44
**Race**												
White	Ref		Ref		Ref		Ref		Ref		Ref	
Hispanic	0.38 (0.36)	0.30	0.03 (0.39)	0.95	−0.87 (1.83)	0.63	−0.80 (1.99)	0.69	0.45 (0.37)	0.23	0.09 (0.40)	0.83
Non-Hispanic Black	1.17 (0.34)	**0.01**	0.69 (0.40)	0.09	0.28 (1.74)	0.87	0.05 (1.85)	0.98	1.22 (0.35)	**0.01**	0.72 (0.42)	0.08
Non-Hispanic Asian and other	0.24 (0.46)	0.59	0.24 (0.47)	0.60	−1.04 (2.07)	0.62	−0.75 (2.13)	0.73	0.32 (0.48)	0.50	0.35 (0.48)	0.46
**Marital status**												
Not single	Ref		Ref		Ref		Ref		Ref		Ref	
Single	1.28 (0.23)	**0.01**	0.82 (0.26)	**0.01**	1.16 (0.79)	0.14	0.66 (0.91)	0.45	1.30 (0.24)	**0.01**	0.83 (0.28)	**0.01**
**Insurance**												
Not Private	Ref		Ref		Ref		Ref		Ref		Ref	
Private	−0.97 (0.21)	**0.01**	−0.51 (0.25)	**0.04**	−0.43 (0.68)	0.53	−0.09 (0.77)	0.91	−0.03 (0.22)	**0.01**	−0.58 (0.26)	**0.03**
**Employment**												
Employed	Ref		Ref		Ref		Ref		Ref		Ref	
Part time	0.57 (0.46)	0.22	0.70 (0.47)	0.14	−0.77 (1.42)	0.59	−0.56 (1.55)	0.72	0.74 (0.49)	0.13	0.83 (0.50)	0.10
Student	0.36 (0.44)	0.42	0.01 (0.46)	0.99	1.14 (1.14)	0.32	1.44 (1.22)	0.24	0.24 (0.48)	0.62	−0.17 (0.50)	0.73
Not Employed	0.80 (0.23)	**0.01**	0.52 (0.25)	**0.04**	1.40 (0.73)	0.06	1.49 (0.79)	0.06	0.73 (0.25)	**0.01**	0.39 (0.26)	0.14

*p*-value < 0.05 are in bold font.

## Data Availability

The demographic and individual level data presented in this study are available on request from the corresponding author due to privacy and ethical reasons. However, environmental-level variables presented in this study were sourced from the Chicago Health Atlas and are available in UIC Box folder at https://uofi.app.box.com/folder/239735247749 (accessed on 25 April 2024). These data were derived from the following resources available in the public domain: “https://chicagohealthatlas.org/ (accessed on 25 April 2024).”

## Supplementary Materials

The following supporting information can be downloaded at: https://www.mdpi.com/article/10.3390/ijerph22111727/s1, Table S1: Environmental-level variables from the Chicago Health Atlas with year and description.
