# Supplementary material for: Environmental Stressors, Anemia, and Depressive Symptoms in Pregnancy: Unpacking the Combined Risks
Source: Int J Environ Res Public Health. 2025 Nov 15;22(11):1727. doi: 10.3390/ijerph22111727 (PMC12652308; doi:10.3390/ijerph22111727)
Supplement: Supplementary file 1 [file ijerph-22-01727-s001.zip › ijerph-3911171-supplementary.pdf]

Table S1: Environmental-level variables from the Chicago Health Atlas with year and description.

Number	Name	Year	Description
1	Robbery (crimes)	2018-2022	Number of robbery is defined as the taking or attempting to take anything of value under confrontational circumstances from the control, custody, or care of another person by force or threat of force or violence and/or by putting the victim in fear of immediate harm.
2	Criminal sexual assault	2017-2021	Number of criminal sexual assault is defined as any sexual act directed against another person, forcibly and/or against that person's will or not forcibly or against the person's will in instances where the victim is incapable of giving consent, including and especially rape.
3	Traffic crashes	2021	Number of crashes on city streets under the jurisdiction of Chicago Police Department.
4	Violent crime (crimes)	2018-2022	Number of crimes related to violence. Includes homicide, criminal sexual assault, robbery, aggravated assault, and aggravated battery.
5	Smoking during pregnancy	2010-2023	Number of births where the mother reported smoking any cigarettes during pregnancy.
6	Arson (crimes)	2017-2021	Arson crimes. Arson is defined as unlawfully and intentionally damaging or attempting to damage any real or personal property by fire or incendiary device.

7	Aggravated assault/battery (crimes)	2018-2022	Aggravated assault and aggravated battery crimes. Aggravated assault is an unlawful attack by one person upon another, wherein the offender displays a weapon in a threatening manner. Aggravated battery is the physical attack itself, wherein the offender uses a weapon or the victim suffers obvious severe or aggravated bodily injury involving apparent broken bones, loss of teeth, possible internal injury, severe laceration, or loss of consciousness.
8	Homicide (crimes)	2018-2022	First and second degree murder cases.
9	Property crime (crimes)	2018-2022	Property crimes. Includes burglary, larceny, motor vehicle theft, and arson crimes.
10	Drug abuse (crimes),	2018-2022	Drug abuse crimes. Drug abuse is defined as the violation of laws prohibiting the production, distribution, and/or use of certain controlled substances and the equipment or devices utilized in their preparation and/or use. While not defined as a major crime, drug abuse rates are included here to show policing and enforcement patterns that may be disproportionate to the true underlying rate of use by different populations.

11	Hardship index	2015-2019	The Hardship Index is a composite score reflecting hardship in the community (higher values indicate greater hardship). It incorporates unemployment, age dependency, education, per capita income, crowded housing, and poverty into a single score that allows comparison between geographies. It is highly correlated with other measures of economic hardship, such as labor force statistics, and with poor health outcomes. See technical notes for details.
12	Social vulnerability index	2018	The Social Vulnerability Index was created to help public health officials and emergency response planners identify and map the communities that will most likely need support before, during, and after a hazardous event, such as a natural disaster, disease outbreak, or chemical spill. SVI indicates relative vulnerability by ranking places on 15 social factors, including unemployment, minority status, and disability, and combining the rankings into a single scale from the 0th percentile (lowest vulnerability) to 100th percentile (highest vulnerability).
13	Unemployment rate	2016- 2020	Percent of residents 16 and older in the civilian labor force who are actively seeking employment.
14	Food stamps	2018-2022	Percent of households receiving Supplemental Nutrition Assistance Program (SNAP) benefits, formerly known as food stamps, over the past 12 months.
15	Rent burdened	2016-2020	Percentage of renter-occupied housing units Households spending more than 30% of income on rent are considered rent-burdened. Rent costs do not

			include utilities, insurance, or building fees.
16	Traffic intensity	2020	Distance-weighted vehicles. A measure of proximity to vehicle traffic, defined as the annual average of the daily count of vehicles within 500 meters, divided by their distance in meters. Higher values indicate higher exposure to heavy traffic.
17	Easy access to fruits and vegetables	2016-2018	Number of adults who reported that it is very easy for them to get fresh fruits and vegetables.
18	Neighborhood safety	2016-2018	Number of adults who report that they feel safe in their neighborhood "all of the time" or "most of the time".
19	College graduation rate	2016-20	Percent of residents 25 or older with a four-year college (bachelor's) degree or higher
20	Per capita income	2017-2021	Income in the past 12 months.
21	Crowded housing	2016-2020	Percent of occupied housing units with more than one occupant per room (e.g. three occupants in a one-bedroom apartment)
22	Tree canopy	2017	Percent of Community Area shaded with tree foliage
23	Particulate matter (PM 2.5) concentration	2020	Annual average concentration in micrograms per cubic meter. PM 2.5, or particulate matter smaller than 2.5 microns in diameter, is one of the most dangerous pollutants because the particles can penetrate deep into the alveoli of the lungs.
24	Food insecurity	2018	Percentage of the population experiencing food insecurity at some point. Food insecurity is the household-level economic and social condition of limited or uncertain access to adequate food, as represented in USDA food-security reports. 2020 data is a

			projection based on 11.5% national unemployment and 16.5% national poverty rate.
25	Eviction rate	2016	Percentage of renter-occupied housing units with an eviction over the past year. An eviction happens when a landlord expels people from property he or she owns. Evictions are landlord-initiated involuntary moves that happen to renters. This is based on available eviction records and estimates for missing data, and does not include voluntary move-outs or evictions that take place outside of the legal system. A high eviction rate could be based on a high number of evictions, a very low number of renter-occupied units, or both.
26	Low food access	2019	Percent of residents who have low access to food, defined solely by distance: further than 1/2 mile from the nearest supermarket in an urban area, or further than 10 miles in a rural area.
27	Lead poisoning	2016	Percent of children ages 1-5 with blood lead level at or above 10 micrograms per deciliter
28	Larceny (theft) (crimes)	2018-2022	Number of crimes Larceny crimes. Larceny is defined as the unlawful taking, carrying, leading, or riding away of property from the possession or constructive possession of another person.
